# Standardisation of rates using logistic regression: a comparison with the direct method

**DOI:** 10.1186/1472-6963-8-275

**Published:** 2008-12-29

**Authors:** Andrea K Roalfe, Roger L Holder, Sue Wilson

**Affiliations:** 1 Primary Care Clinical Sciences, School of Health and Population Sciences, University of Birmingham, Edgbaston, Birmingham, B15 2TT, UK

## Abstract

**Background:**

Standardisation of rates in health services research is generally undertaken using the direct and indirect arithmetic methods. These methods can produce unreliable estimates when the calculations are based on small numbers. Regression based methods are available but are rarely applied in practice. This study demonstrates the advantages of using logistic regression to obtain smoothed standardised estimates of the prevalence of rare disease in the presence of covariates.

**Methods:**

Step by step worked examples of the logistic and direct methods are presented utilising data from BETS, an observational study designed to estimate the prevalence of subclinical thyroid disease in the elderly. Rates calculated by the direct method were standardised by sex and age categories, whereas rates by the logistic method were standardised by sex and age as a continuous variable.

**Results:**

The two methods produce estimates of similar magnitude when standardising by age and sex. The standard errors produced by the logistic method were lower than the conventional direct method.

**Conclusion:**

Regression based standardisation is a practical alternative to the direct method. It produces more reliable estimates than the direct or indirect method when the calculations are based on small numbers. It has greater flexibility in factor selection and allows standardisation by both continuous and categorical variables. It therefore allows standardisation to be performed in situations where the direct method would give unreliable results.

## Background

Standardisation is frequently used in medical research to allow for the influence of differences in case mix (such as different age or sex distributions) when comparing populations or sub-groups (such as different regions or hospitals).

The indirect arithmetic method is the most commonly used standardisation method in the literature. It compares the actual number of events in a local area (e.g. Birmingham) with the number expected when factor-specific event rates (e.g. age, sex) in a reference population (e.g. England) are applied to the local population. This method is often used to look at differences in mortality rates by means of standardised mortality ratios (SMRs) [1,2]. It has also been used to assess other events such as NHS performance indicators [3,4]. However ratios cannot be directly compared to one another with this method only to the Standard (For example SMR = 100). In addition, indirect standardisation cannot be applied if the number of events in the reference population is unknown.

Direct standardisation, another frequently used method, involves applying local age-sex specific rates to the age-sex population estimate of a reference, or standard [5-7]. This approach enables comparisons between local areas, for example, comparing the incidence of cancer in different regions of England, and allows for the differing age and gender structures in different areas of the country [8]. This technique therefore depends on the availability of age/sex specific rates for a local population.

For relatively rare conditions, there will be considerable instability in local age/sex-specific rates of disease and indirect standardisation is a more robust method if the populations are small or there is uncertainty about the stability of age-specific death rates [9].

Logistic regression standardisation, an alternative to the arithmetic methods has advantages over these latter approaches when individual level data are available, through for example, a survey.

Logistic regression allows the effect of variables (e.g. age and sex), and interactions between these factors, on outcomes of interest (e.g. presence of disease) to be estimated. Additional demographic data may be of use and also variables, such as age, could be included as continuous variables in the model, thus having a smoothing effect on the estimates.

Using Poisson regression to model rates and adjust for confounders is not uncommon,[10] however such modelling does not usually apply a standard population to the models identified. Standardisation using logistic regression modelling involves calculating the sum of the predicted probabilities of the outcome of interest for each individual in the local population and establishing the ratio of the observed and expected event rates [11]. Examples of the use of regression standardisation include describing variation in practice admission rates [12]; measuring income related quality of life [13]; measuring inequity in the delivery of healthcare [14]; and calculating hospital mortality ratios, adjusting for age, sex, diagnosis, admission method and length of stay [15,16].

The equivalence of indirect and logistic regression-based standardisation with a saturated model when adjusting for case-mix has been previously demonstrated [11]. Nevertheless, the arithmetic direct/indirect methods continue to be the more popular and widely utilised methods employed in health service research. The most probable reasons for this may be the lack of survey data and the perception that logistic regression-based standardisation is more difficult than the arithmetic methods. This paper aims to illustrate the application of logistic regression to calculate standardised smoothed prevalence estimates of disease when the direct method may produce biased estimates and the indirect method is not possible.

### Illustrative data

The Birmingham elderly thyroid study (BETS), a cross-sectional survey of people aged 65 years and over has been used to illustrate the methods discussed in this paper. BETS aim was to determine the prevalence of subclinical hypothyroidism and hyperthyroidism in the elderly [17]. Demographic data were collected from participants and included age and sex. Of the 16,125 patients invited to participate in BETS, only 5,881 (36.5%) took part in the survey. Response rates varied by age (43% 65–69 years to 26% 80+ years) and gender (35% male vs. 40% female). Participants had a different age and sex structure to that in the National population and adjustment was necessary to allow inferences about the prevalence of disease in England and Wales to be made. A standardisation approach was chosen to correct for this response bias [9].

The crude prevalence of subclinical hyperthyroidism and subclinical hypothyroidism were 2.2% (128/5881) and 2.9% (168/5881) respectively. Age-specific subclinical hyperthyroidism rates ranged from 1.7% (16/945) in males aged 65–69 years to 2.3% (9/388) in males aged 80+.

## Methods

To calculate rates for subclinical hyperthyroidism standardised by age and gender by the direct method, ages were categorised into four 5-year age bands (65–69, 70–74, 75–79, 80 and over). The formulae used to calculate the standardised rates are given below:

### (i) Direct method

The directly standardised rate is obtained by dividing the total expected number of cases in a standard population by the standard population size

(1.1) $$\text{Standardised rate}=\frac{\sum_{\text{ij}}{\text{N}}_{\text{ij}}{\hat{\text{p}}}_{\text{ij}}}{\text{N}}$$

where i = 1 to 4 age groups and j = 1, 2 sexes, N_ij _is the standard population size in age group i, sex j, $\sum_{\text{ij}}{\text{N}}_{\text{ij}}=\text{N}$, p_ij _is the age-sex specific rate in the study, ${\hat{p}}_{ij}$ is the estimated age-sex specific rate in the study, n_ij _is the age-sex specific population in the study.

The standard error of a directly standardised rate is given by:

(1.2) $$\text{standard error}\left(\text{standardised rate}\right)=\frac{\sqrt{\sum_{\text{ij}}{\text{N}}_{\text{ij}}^{2}\frac{{\text{p}}_{\text{ij}}(1-{\text{p}}_{\text{ij}})}{{\text{n}}_{\text{ij}}}}}{\text{N}}$$

Where p_ij _are all small, as is often the case, p_ij _(1-p_ij_) can be replaced with p_ij _thus [1.2] reduces to

(1.3) $$\text{standard error}\left({\text{standardised rate}}_{\;}\right)=\frac{\sqrt{\sum_{\text{ij}}{\text{N}}_{\text{ij}}^{2}\frac{{\text{p}}_{\text{ij}}}{{\text{n}}_{\text{ij}}}}}{\text{N}}$$

A 95% confidence interval for the standardised rate (using a normal approximation) is then:

standardised rate ± 1.96 (standard error (standardised rate))

### (ii) Logistic regression method

When individual data (presence/absence of disease, age and sex) are available, logistic regression allows us to examine the relationship between the probability of disease (p) and potential explanatory variables via the logit transformation of p:

(2.1) $$\text{Logit}={\text{log}}_{\text{e}}\left(\frac{\text{p}}{\left(1-\text{p}\right)}\right)=\alpha +\beta (\text{age})+\gamma (\text{sex})+\beta_{\gamma }(\text{age})(\text{sex})$$

where p is the age-sex specific rate in the study, α, β, γ and β_γ _are unknown parameters, age (years), sex (1 = male, 0 = female)

The data can be used to provide estimates (maximum likelihood) of these parameters and hence an estimated $\text{logit}=\hat{\alpha }+\hat{\beta }(\text{age})+\hat{\gamma }(\text{sex})+{\hat{\beta }}_{\gamma }(\text{age})(\text{sex})$

The estimated logit is then weighted by the Standard age/sex specific population sizes (N_agesex_)

(2.2) $$\text{standardised logit}=\frac{\sum_{\text{agesex}}{\text{N}}_{\text{agesex}}(\text{estimated logit})}{\text{N}}$$

where N_agesex _is the population with a specific age and sex $\sum_{\text{agesex}}{\text{N}}_{\text{agesex}}=\text{N}$.

The standardised rate is then obtained by back transformation:

(2.3) $$\text{standardised rate}=\frac{\exp(\text{standardised logit})}{1+\exp(\text{standardised logit})}$$

The variance of the standardised logit is given by:

(2.4) $$\text{variance }(\text{standardised logit})=\frac{\sum_{\text{agesex}}{\text{N}}_{\text{agesex}}^{2}\mathrm{var}(\text{estimated logit})}{{\text{N}}^{2}}$$

and standard error of the standardised logit is thus:

(2.5) $$\text{standard error }(\text{standardised logit})=\frac{\sqrt{\sum_{\text{agesex}}{\text{N}}_{\text{agesex}}^{2}{\left(\text{standard error }\left(\text{estimated logit}\right)\right)}^{2}}}{\text{N}}$$

The 95% confidence interval of the standardised logit is: standardised logit ± 1.96 standard error (standardised logit) = (lower, upper)

Back transforming again to obtain the confidence interval for the standardised rate:

(2.6) $$95\%\text{ confidence interval standardised rate}=\left(\frac{\exp(\text{lower})}{1+\exp(\text{lower})},\frac{\exp(\text{upper})}{1+\exp(\text{upper})}\right)$$

This method of calculating the confidence interval for the standardised logit and then back transforming to obtain standardised rates is used since the distribution of the logit is liable to be closer to the Normal distribution since the scale ranges from (-∞ to +∞) as opposed to between (0 and 1). The price for this benefit is that the estimator is a biased estimator of the statistic in equation 1.1. The bias could be estimated by using equation 1.1 where ${\overset{⌢}{p}}_{ij}$ is obtained by back transforming the logits.

As with any logistic model building process, the linearity assumption for any continuous variables should be confirmed. A method based on quartiles can be used to test this assumption. A categorical variable with 4 levels is created using three cutpoints based on the quartiles of the distribution of the continuous variable (e.g. age). The model can then be refitted with the categorical variable and a plot of the estimated coefficients versus the midpoints of the quartile groups can be examined to determine linearity [18]. The effectiveness of the model to describe the outcome variable should also be assessed with the Hosmer-Lemeshow goodness of fit test [18].

## Results

### Illustrative example – calculating age and sex standardised rates

#### Direct method

To obtain the directly standardised prevalence rate, BETS data were categorised and applied to the National population [19]. Table 1 provides a breakdown of these populations and the calculations involved.

**Table 1:** Direct standardisation calculations for subclinical hyperthyroidism

Age group	Sex	Cases in study (r_ij_)	Age-sex distribution of the study population (n_ij_)	Age-sex specific prevalence rate in study (per 100) $\left({\hat{p}}_{ij}=\frac{r_{ij}}{n_{ij}}\times 100\right)$	Age-sex distribution of E & W in 100's (N_ij_)	Expected cases *N*_*ij *_× ${\hat{p}}_{ij}$	$\frac{N_{ij}^{2}\times {\hat{p}}_{ij}}{n_{ij}}$
65–69	male	16	945	1.6931	11306	19142	22902048
65–69	female	13	981	1.3252	12149	16100	19938220
70–74	male	14	916	1.5284	9541	14582	15189849
70–74	female	14	839	1.6687	11228	18736	25073151
75–79	male	17	643	2.6439	7334	19390	22116112
75–79	female	29	660	4.3939	9865	43346	64789451
80+	male	9	388	2.3196	7791	18072	36288203
80+	female	17	509	3.1434	15327	48179	145077055
							
Total		128	5881	2.1765	84541	197547	351373093

From [1.1] standardised $\text{rate}=\frac{\sum_{\text{ij}}{\text{N}}_{\text{ij}}{\hat{\text{p}}}_{\text{ij}}}{\text{N}}=\frac{197547}{84541}=2.337$ per 100 population

Using [1.3] and substituting ${\hat{\text{p}}}_{\text{ij}}$ for p_ij_

$$95\%\text{ confidence interval}=\text{standardised rate}\pm 1.96\frac{\sqrt{\sum_{\text{ij}}{\text{N}}_{\text{ij}}^{2}\frac{{\hat{\text{p}}}_{\text{ij}}}{{\text{n}}_{\text{ij}}}}}{\text{N}}$$

$$95\%\text{ confidence interval}=2.337\pm 1.96\frac{\sqrt{351373}093}{84541}=(1.90,2.77)$$

#### Logistic method

The logistic regression analysis used disease (1 = disease present; 0 = absent) as the dependent binary variable and age (continuous), sex, and the interaction of age and sex as independent variables. Logistic regression software packages either automatically set up categorical variables as class variables, or enable the creation of dummy variables (e.g. sex (1 = male, 0 = female)) with interaction terms being the corresponding products of variables.

The resultant logistic regression model for subclinical hyperthyroidism was:

Logit = - 7.2175 + 0.0461 age + 1.0337 sex - 0.0152 age*sex

Age was found to be linearly related to the logit. The interaction term was not significant in this model however it has been left in for illustrative purposes. Logits for all unique combinations of age and sex were then estimated from this model (e.g. a male aged 65: Logit = -7.2175 + (0.0461*65) +1.0337 - (0.0152*65) = -4.18) and weighted by the corresponding standard population size (National population estimates were available from the Office for National Statistics by gender and single year of age). This was implemented by creating a dataset containing the study data plus an additional 52 'dummy' records, one for each unique combination of single year of age and sex variables but with the outcome defined as missing. The logistic regression was run with the default set up of variables and variables interactions, the resulting model being based only on the study data for which there was outcome data available (Table 2). A new output dataset containing the logits and standard error (logits) was then generated by the logistic procedure (SAS) for all observations in the input dataset. The 52 'dummy' records were then extracted from this output file (Table 3) and merged with the corresponding age-sex specific standard population estimate (Table 4) to enable the following weighting calculations:

**Table 2:** Logistic regression model for subclinical hyperthyroidism

Parameter	DF	Estimate	Error	Chi-Square	Pr > ChiSq
Intercept	1	-7.2175	1.1495	39.4248	< .0001
age	1	0.0461	0.0154	8.9420	0.0028
sex	1	1.0337	1.1495	0.8087	0.3685
age*sex	1	-0.0152	0.0154	0.9796	0.3223

**Table 3:** Predicted probabilities and logits for subclinical hyperthyroidism

Obs	Age	Sex	logit	selogit	varlogit
1	65	male	-4.18064	0.24818	0.06159
2	65	female	-4.26593	0.23466	0.05506
3	66	male	-4.14982	0.22861	0.05226
4	66	female	-4.20461	0.21801	0.04752
5	67	male	-4.11900	0.20990	0.04405
6	67	female	-4.14330	0.20189	0.04076
7	68	male	-4.08818	0.19231	0.03698
8	68	female	-4.08199	0.18645	0.03476
.	.	.	.	.	.
51	90+	male	-3.41019	0.40868	0.16702
52	90+	female	-2.73314	0.31215	0.09744

**Table 4:** Logistic regression standardisation calculations for subclinical hyperthyroidism

Age group	Sex	Logit_ij_	SE(Logit_ij_)	Age-sex popn E & W in 1000's (N_ij_)	N_ij _× Logit_ij_	N_ij_^2 ^× (SE (logit_ij_))^2^
65	male	-4.181	0.248	243.5	-1093.1	3920.2
65	female	-4.266	0.235	256.6	-848.3	1947.4
66	male	-4.150	0.229	235.7	-1041.3	3158.0
66	female	-4.205	0.218	250.4	-827.1	1572.1
67	male	-4.119	0.210	226.9	-986.3	2499.2
67	female	-4.143	0.202	243.8	-804.5	1256.9
68	male	-4.088	0.192	218.4	-933.8	1965.9
68	female	-4.082	0.186	206.1	-779.4	994.3
.						
90+	female	-2.733	0.312	290.7	-889.4	6774.6
Total				8454.1	-31561.0	57911.1

$$\text{Applying [2}\text{.2] standardised logit}=\frac{-31561.0}{8454.1}=-3.733$$

$$\text{Using [2}\text{.5] standard error (standardised logit)}=\sqrt{\frac{57911.1}{8454.1^{2}}}=0.0285$$

95% confidence interval for the standardised logit = standardised logit ± 1.96 standard error (standardised logit)

-3.733 ± 1.96 (0.0285) = (-3.789, -3.677)

The standardised rate and confidence interval are then obtained using [2.3] and [2.6] respectively.

$$\text{standardised rate}=\frac{\exp(-3.733)}{1+\exp(-3.733)}=\frac{0.024}{1.024}=0.0234=2.34\text{ per }100\text{ persons aged }65+$$

$$95\%\text{ Confidence interval}=\left(\left(\frac{\exp(-3.789)}{1+\exp(-3.789)}\right),\left(\frac{\exp(-3.677)}{1+\exp(-3.677)}\right)\right)=(2.21,2.47)$$

Table 5 summarises the overall and sex-specific standardised prevalence estimates obtained from both methods for subclinical disease. The rates were similar in magnitude for both methods, however the confidence intervals produced by the logistic method were narrower.

**Table 5:** Comparison of standardised rates using direct and logistic regression approaches

Disease	Sex	Direct^1^	Logistic^2^
subclinical hyperthyroidism	Male	1.98 (1.44, 2.51)	1.98 (1.82, 2.14)
	Female	2.60 (1.96, 3.25)	2.64 (2.46, 2.84)
	Total	2.34 (1.90, 2.77)	2.34 (2.21, 2.47)
			
subclinical hypothyroidism	Male	2.19 (1.61, 2.77)	2.08 (1.92, 2.26)
	Female	3.66 (2.92, 4.41)	3.65 (3.43, 3.89)
	Total	3.04 (2.54, 3.53)	2.88 (2.74, 3.02)

^1 ^standardised by age group and sex

^2 ^standardised by age and sex

## Discussion

This study has illustrated the similarity of standardised rates when calculated by direct standardisation and logistic regression and has demonstrated the value of logistic regression in instances where individual level data are available.

Logistic regression is a practical and intuitive approach to standardisation. Most statistical packages contain regression analysis procedures and the methods described in this paper are suitable for implementation in SAS and STATA (SPSS requires an additional step to obtain case-wise estimates of logit and standard error (logit) [20]).

Direct standardisation requires categorisation of the population and the rates. If adjustment is necessary for several variables (such as age, sex and deprivation) then some categories may have very low or zero rates, thus generating an imprecise estimate of the standardised rate. Once direct standardisation has been implemented, then calculation of rates is generally a routine method (requiring only the input of category specific numbers of cases) and the potential bias caused by small numbers may be missed. Logistic regression standardisation tends to fail to converge to a solution when the number of cases are too small, alerting the researcher to problems with the data.

The main advantage of the logistic regression method is that it allows adjustment by continuous variables in addition to categorical variables and therefore has the potential to lose less information than the direct method which only allows for standardisation by categorical variables. The allowance of continuous variables also has a beneficial smoothing effect on the model. Logistic regression standardisation can also allow for adjustment by non-linear variables and interactions between variables. The structure of the model can be extended to include random effects [21]. This may be particularly useful when allowing for clustering effects (e.g. hospitals, general practices), thereby incorporating cluster variation in the standard error of the predicted values. The logistic regression method also allows standardisation when there is missing data through the process of imputation whereas the direct method would exclude these observations from the analysis [22]. In addition this method will identify the amount of variation explained by the variables and will highlight those that have a significant effect on the outcome, giving the analyst the choice to include or exclude variables [18]. Nevertheless, to avoid the problem of data dredging any potential variables should be decided on prior to analysis being performed [23].

Another possible benefit of logistic regression standardisation is that the method may identify the absence of significant variables and consequently demonstrate that there is no requirement or benefit from standardisation.

## Conclusion

Logistic regression based standardisation is a practical alternative to the direct method. It produces more dependable estimates than the direct method when there are small numbers involved. It has greater flexibility in factor selection and allows standardisation by both continuous and categorical variables. It also has the benefit of a smoothing property when including continuous variables. The method allows standardisation to be performed where the direct method would give unreliable results.

## Competing interests

The authors declare that they have no competing interests.

## Authors' contributions

AR and RH planned the study. AR conducted the analyses and produced the first draft of the manuscript. RH provided guidance on statistical analysis. All authors contributed to the reviewing and editing of the manuscript. All authors read and approved the final manuscript.

## Pre-publication history

The pre-publication history for this paper can be accessed here:


